# Supplementary material for: Hyperoxia Reprogrammes Microvascular Endothelial Cell Response to Hypoxia in an Organ-Specific Manner
Source: Cells. 2022 Aug 9;11(16):2469. doi: 10.3390/cells11162469 (PMC9406746; doi:10.3390/cells11162469)
Supplement: Supplementary file 1 [file cells-11-02469-s001.zip › cells-1807881-SM.pdf]

Figure S1

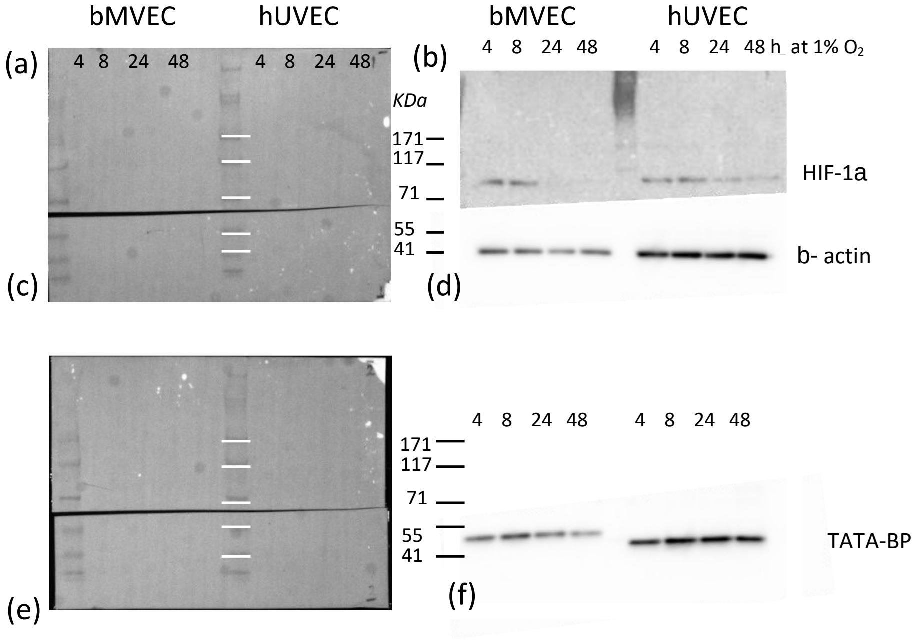

Validation of β-actin as loading control for nuclear extracts of endothelial cells. Two gels were loaded with the same nuclear protein extracts of endothelial cells (prepared in same tube, loaded ½ in each gel), from human (hUVEC) and murine (*b*MVEC) origin; gels were cut just below the mw standard of 71 KDa, to split between high and low molecular weight proteins; the top half of gel 1 (a) was probed for HIF-1a (b), the bottom (c) was probed for β-actin, shown in panel (d); the lower portion of the cut blot from gel 2 (e) containing the exact same protein as in (c) and (d), was probed for TATA-binding protein. We show TATA-BP in figure 1(c) to show nuclear protein is present, and subsequently used β-actin because of the more consistent signal across all replicates.

Figure S2

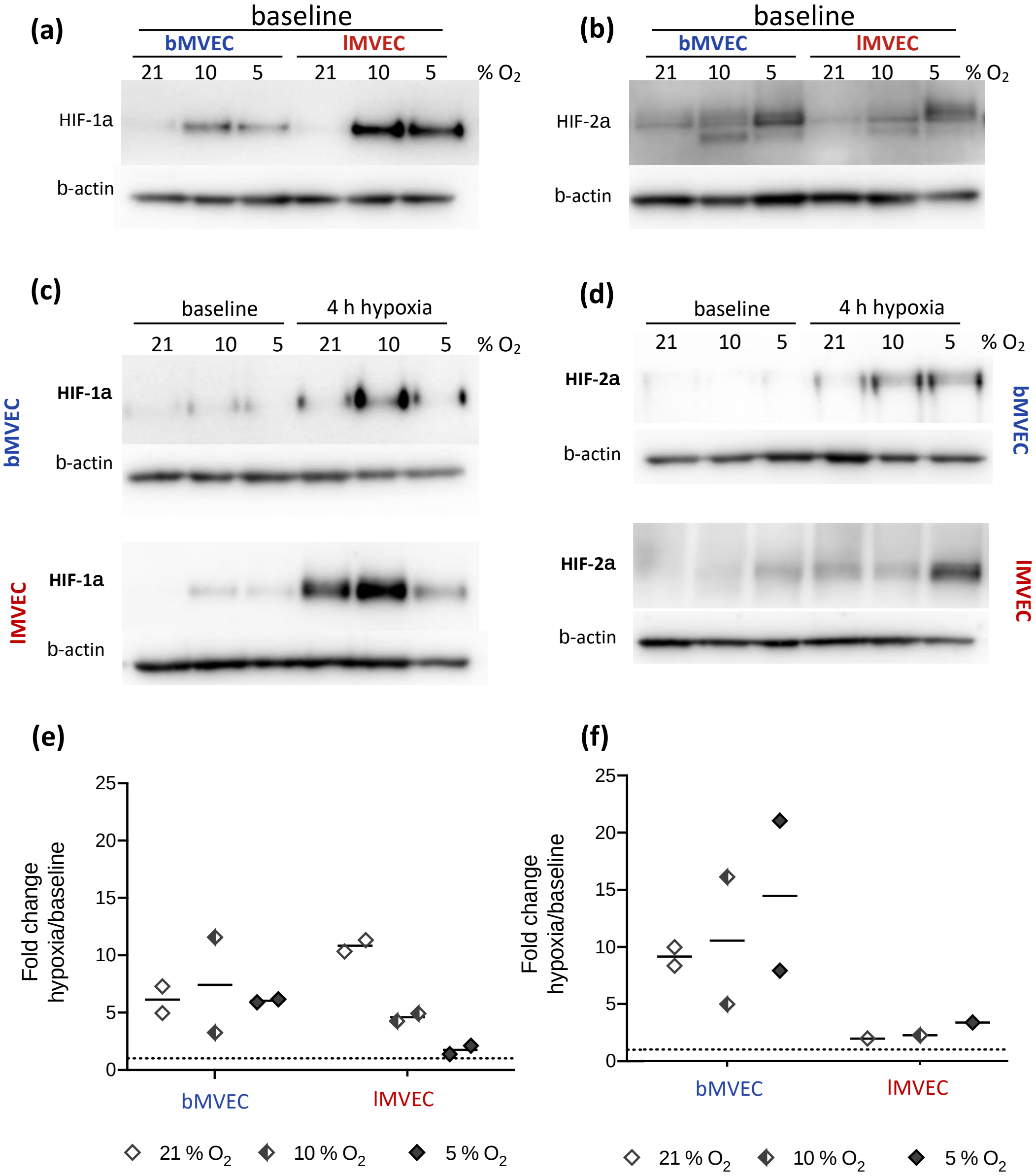

Figure S2: Baseline and change of HIF-α isoform protein levels is shaped by O_2 priming (related to Figure 2)

(a, b) Representative western blot showing HIF-1α (A) and HIF-2α (B) signal in nuclear extracts of brain and lung MVEC grown at different oxygen levels; β-actin is shown as loading control

(d, d) Representative western blot of HIF-1α (C) and HIF-2α (D) signal at baseline and following 4h of hypoxia, from nuclear extracts of brain and lung MVEC expanded at different oxygen levels

(e) Ratio of 4h hypoxia:baseline HIF-1α signal, normalised to loading control, as displayed in Figure S1C; n=2

(f) Ratio of 4h hypoxia:baseline HIF-2α signal, normalised to loading control, as displayed in Figure S1D; n=2

Figure S3

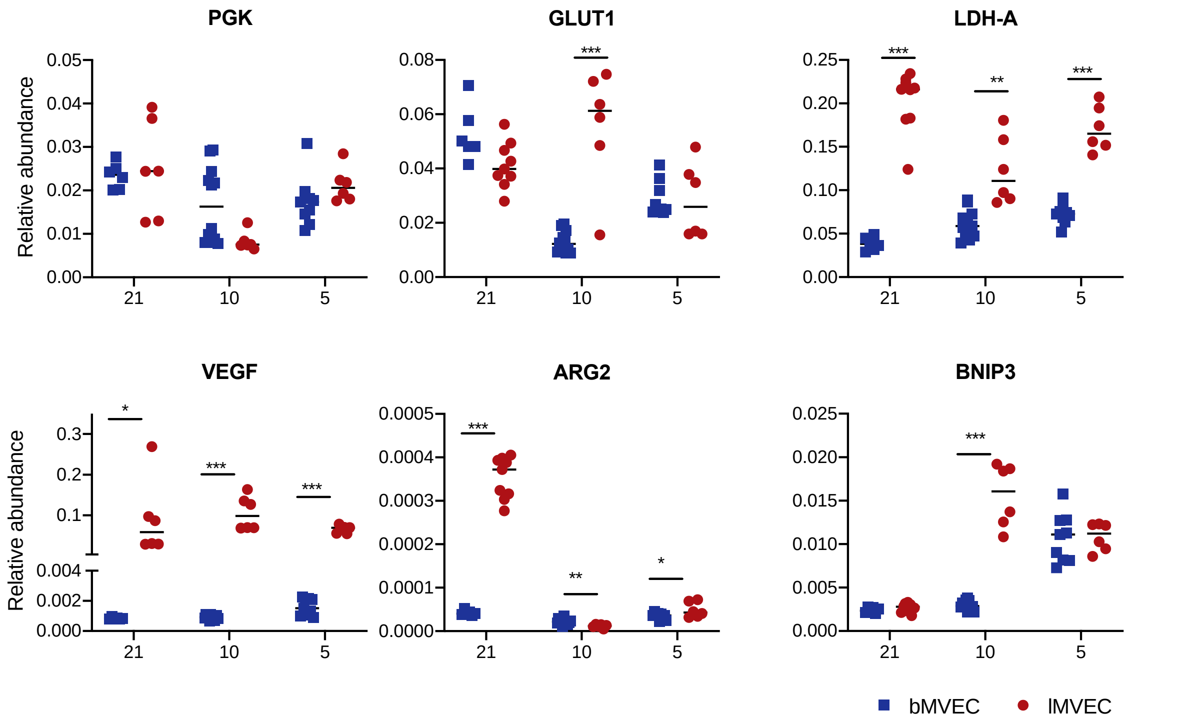

Figure S3: Relative mRNA abundance of hypoxia response genes under different baseline O_2 conditions (Related to Figure 2)

Total RNA was isolated from brain and lung MVECs expanded at different oxygen levels (21, 10 and 5 % O_2). Transcript levels were measured by RT-qPCR and are shown as relative abundance after normalization to the β-actin housekeeping mRNA. n≥2 independent experiments with 3 replicates each; statistical significance was assessed by t-tests corrected for multiple comparisons (Holm-Sidak); *p<0.05, **p<0.01, ***p<0.001

Table S1: Summary of statistical analyses for figure 2F, using 2-way ANOVA with Holm-Sidak's multiple comparison test on the log10 of fold change. *p<0.05, **p<0.005, ***p<0.001, ****p<0.0001. When comparing lung versus brain, asterisks are colored to match the higher value, when significant. Note: Arg2 mRNA was undetectable in baseline *b*MVEC at 21% O_2.

	target	PGK				VEGF				GLUT1			
	time	4h	8h	24h	48h	4h	8h	24h	48h	4h	8h	24h	48h
bMVEC	10 % vs 21 %	****	****	****	****	****	****	****	****	ns	****	****	****
	5 % vs 21 %	****	****	****	****	****	****	****	****	ns	ns	****	****
	10 % vs 5%	ns	*	****	****	ns	****	****	****	ns	****	****	****
lMVEC	10 % vs 21 %	ns	ns	****	****	ns	ns	ns	****	ns	*	ns	****
	5 % vs 21 %	ns	ns	ns	****	ns	**	ns	****	ns	ns	****	****
	10 % vs 5%	ns	***	**	****	ns	***	ns	***	ns	ns	*	ns
lMVEC vs bMVEC	21%	****	***	****	****	**	**	****	*	ns	ns	****	****
	10%	ns	ns	ns	ns	***	****	****	****	ns	****	***	ns
	5%	ns	**	****	**	****	****	*	ns	ns	ns	****	****

	target	ARG2				LDH				BNIP3			
	time	4h	8h	24h	48h	4h	8h	24h	48h	4h	8h	24h	48h
bMVEC	10 % vs 21 %	**	****	****	-	ns	ns	****	****	****	****	ns	*
	5 % vs 21 %	ns	*	****	-	ns	ns	ns	ns	**	****	****	****
	10 % vs 5%	*	****	****	***	ns	ns	****	****	ns	****	****	****
lMVEC	10 % vs 21 %	ns	**	ns	****	**	ns	****	****	ns	*	**	****
	5 % vs 21 %	ns	ns	ns	****	ns	*	ns	ns	ns	ns	**	***
	10 % vs 5%	ns	***	***	ns	****	****	***	****	ns	ns	ns	***
lMVEC vs bMVEC	21%	ns	**	****	-	*	ns	ns	ns	**	****	****	****
	10%	ns	ns	ns	ns	ns	ns	ns	***	ns	****	ns	***
	5%	ns	ns	ns	ns	ns	*	ns	ns	ns	ns	****	ns

Figure S4

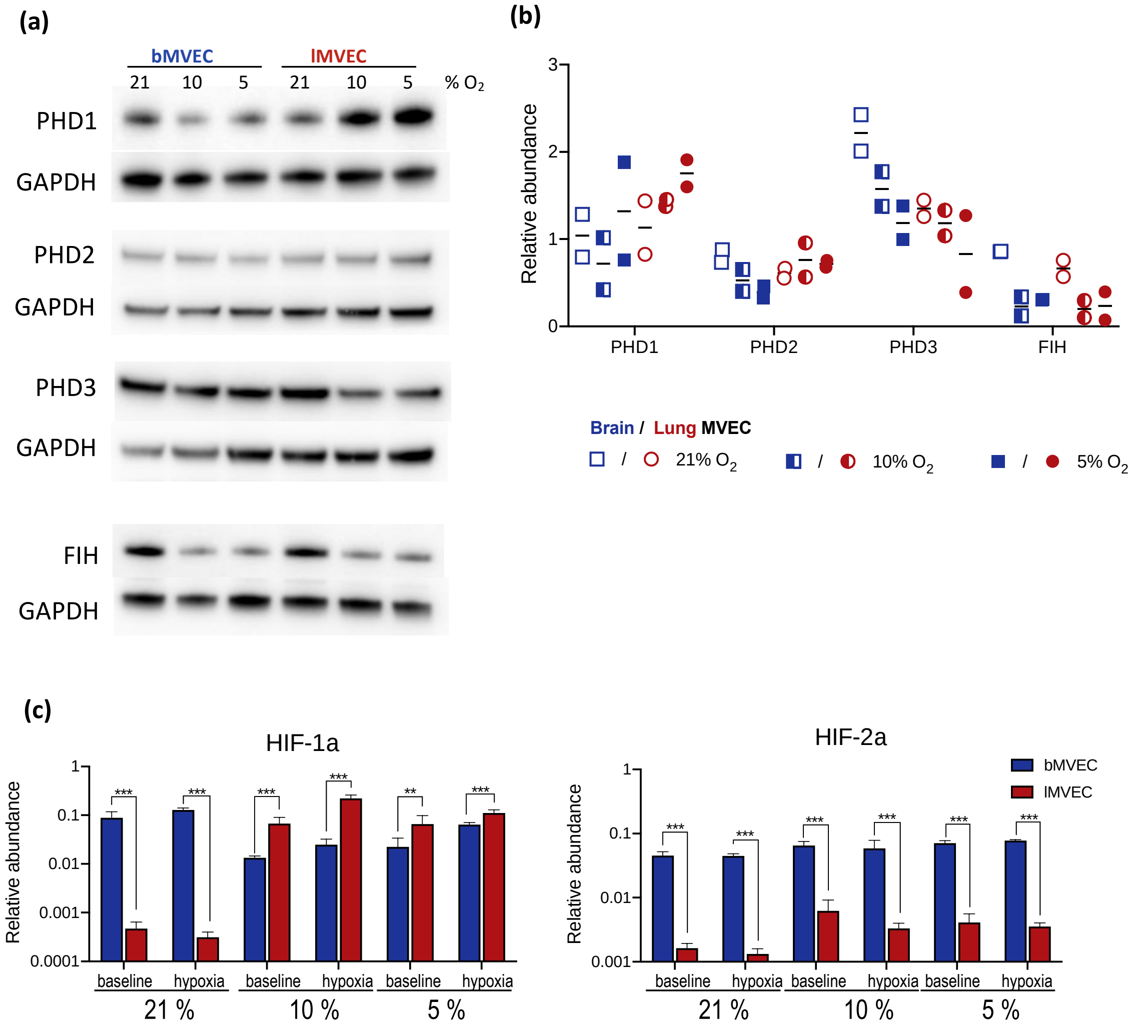

Figure S4: Upstream regulation of HIF-α is mildly affected by O_2 but is not tissue-specific (Related to Figure 2)

(a) Representative image of western blot probed for enzymes responsible for HIF protein stabilization (prolyl hydroxylases, PHD1-3) and HIF transcriptional activity (factor inhibiting HIF, FIH) using whole-cell protein lysate.

(b) Signal from (A) was quantified by densitometry and normalised to loading control, GAPDH. (n=2)

(c) Comparison of HIF-1α and HIF-2α mRNA levels MVEC from lung and brain, expanded in different oxygen levels, at baseline and after 4h at 1% O_2. Transcript levels were quantified by RT-qPCR and are displayed as average ± SD relative abundance compared to the β-actin housekeeping gene (n=3); statistical significance assessed by t-tests corrected for multiple comparisons (Holm-Sidak); $^{**}p<0.01$, $^{***}p<0.001$

Figure S5

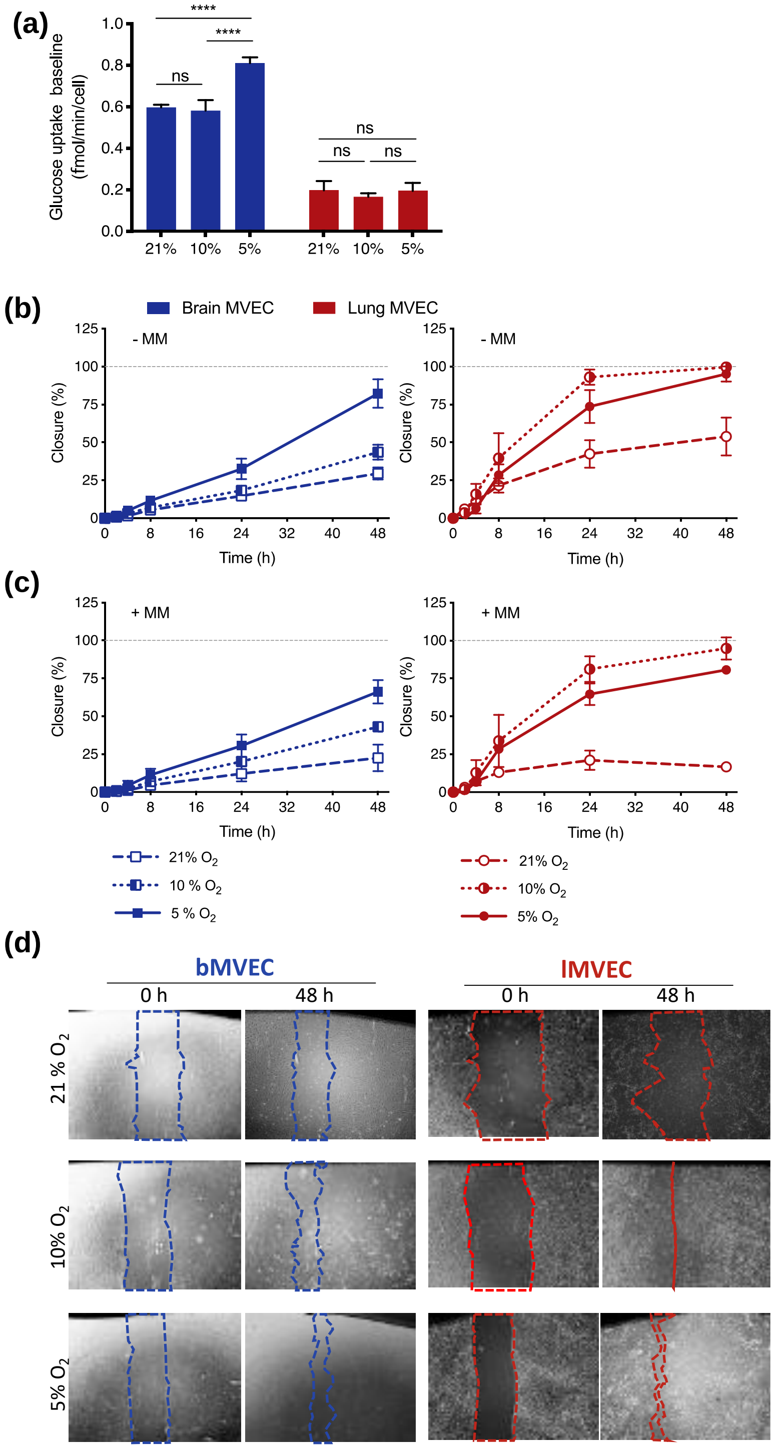

(e)

- MM	bMVEC					
time	0	2	4	8	24	48
21 % vs 10%	ns	ns	ns	ns	ns	***
21% vs 5 %	ns	ns	ns	ns	****	****
10 % vs 5 %	ns	ns	ns	ns	***	****

- MM	lMVEC					
time	0	2	4	8	24	48
21 % vs 10%	ns	ns	ns	****	****	****
21% vs 5 %	ns	ns	ns	ns	****	****
10 % vs 5 %	ns	ns	ns	**	****	****

- MM	Lung vs Brain MVEC					
time	0	2	4	8	24	48
5 %	ns	ns	ns	****	****	***
10 %	ns	ns	***	****	****	****
21 %	ns	ns	ns	***	****	****

+ MM	bMVEC					
time	0	2	4	8	24	48
21 % vs 10%	ns	ns	ns	ns	ns	****
21% vs 5 %	ns	ns	ns	ns	***	****
10 % vs 5 %	ns	ns	ns	ns	ns	****

+ MM	lMVEC					
time	0	2	4	8	24	48
21 % vs 10%	ns	ns	ns	***	****	****
21% vs 5 %	ns	ns	ns	**	****	****
10 % vs 5 %	ns	ns	ns	ns	***	***

+ MM	Lung vs Brain MVEC					
time	0	2	4	8	24	48
5 %	ns	ns	ns	***	****	***
10 %	ns	ns	ns	****	****	****
21 %	ns	ns	ns	ns	ns	ns

Figure S5: Oxygen effects on glucose uptake and cell migration

(a) Baseline glucose uptake was quantified in lung and brain MVEC maintained in different O_2 conditions. Displayed as average ± SD, statistical analysis using 2-way ANOVA with Holm-Sidak's multiple comparison test, ****$p<0.0001$ (Related to Figure 3)

(b c) Wound closure assays were performed using brain and lung MVEC expanded at different O_2 levels; wounds were applied using a P1000 tip at t=0 and images taken at regular intervals. Quantification of wound closure was made in monolayers treated with DMSO (B) or 10 mM Mitomycin C (c) for 2h before the scratch was applied.

(d) Representative images of wells during migration assay in the presence of Mitomycin C at the start and end of assay.

(e) Summary of statistical analyses for data in B,C; 2-way ANOVA. Multiple comparisons using Holm-Sidak correction; **$p<0.005$, ***$p<0.001$, ****$p<0.0001$, n= 4.

Figure S6

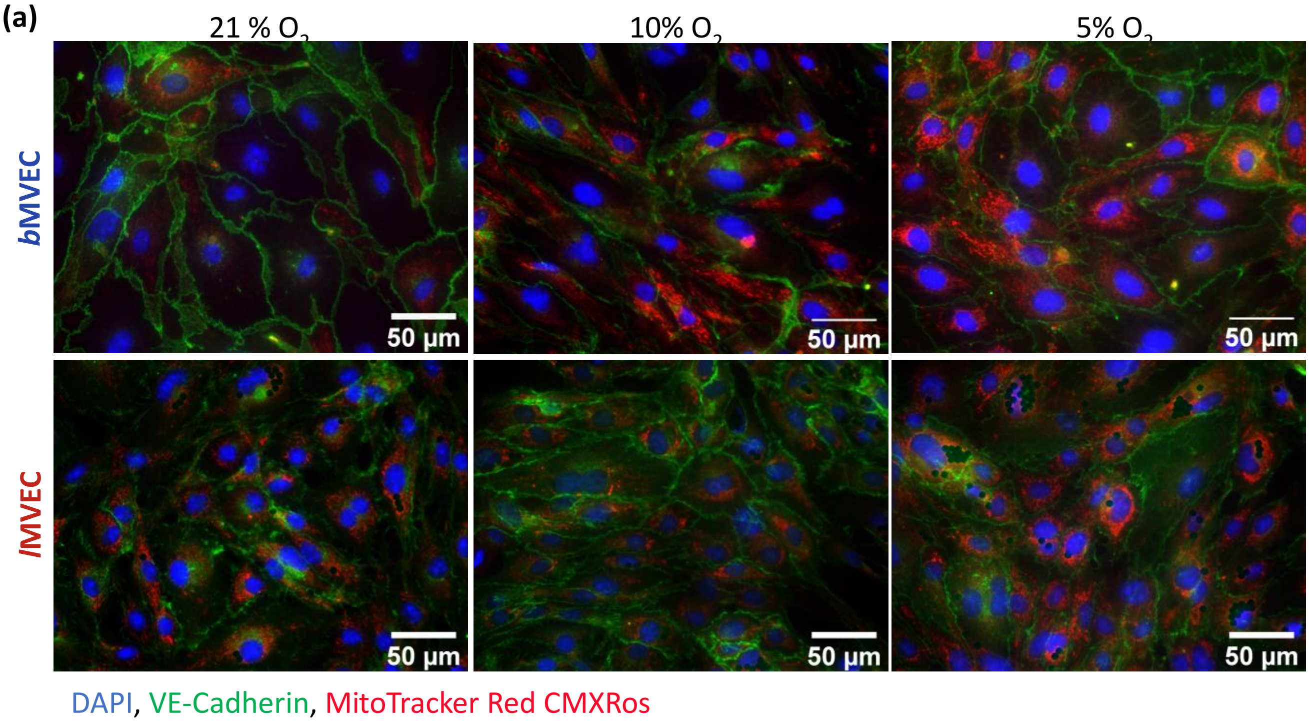

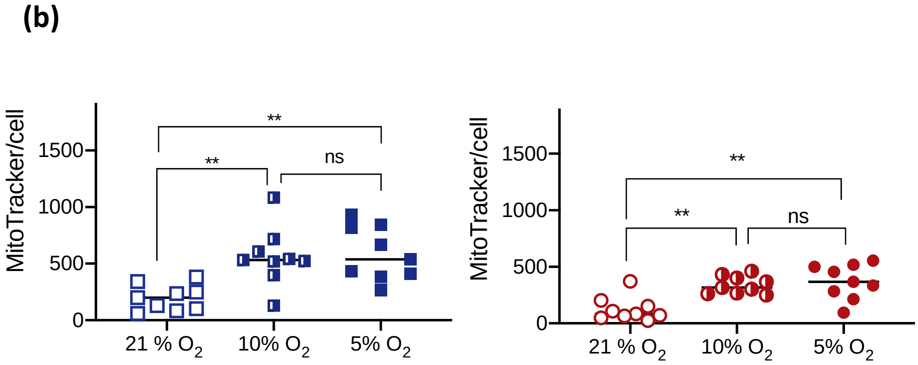

Figure S6: Mitochondrial activity is impaired by excess O_2

(a) Representative images of brain and lung MVEC expanded at different O_2 levels, stained for EC marker VE-Cadherin (green), mitochondrial membrane potential (MitoTracker Red CMXRos, red) and counter-stained with DAPI (blue)

(b) Quantification of the MitoTracker stain shown in (a) for *b*MVEC (left, blue) and *l*MVEC (right, red); different thresholds were used for the two cell populations; n=10 pictures from a single biological control. Statistical analysis using 2-way ANOVA with Holm-Sidak's multiple comparison test (**p<0.005)

Table S2: Summary of Reagents and Resources

REAGENT/RESOURCE	REFERENCE/SOURCE	IDENTIFIER or CATAOGUE NUMBER
Antibodies		
β-actin (clone AC-15)	Sigma-Aldrich	Cat# A1978
CD31 (clone MEC 13.3)	BD Pharmingen	Cat# 553370
FIH (clone D19B3)	Cell Signaling Technology	Cat# 4426S
GAPDH (clone G-9)	Santa Cruz Biotechnology	Cat# sc-365062
HIF-1α	Novus Biologicals	Cat# NB100-449
HIF-2α	R&D Systems	Cat# AF2997
PHD1 (clone EPR2746)	Abcam	Cat# ab113077
PHD2	Novus Biologicals	Cat# NB100-2219
PHD3	Novus Biologicals	Cat# NB100-303
TATA-BP	Abcam	Cat# ab63766
VE-Cadherin	R&D Systems	Cat# AF1002
Goat IgG - HRP conjugated	Santa Cruz Biotechnology	Cat# sc-2304
Mouse IgG - HRP conjugated	Santa Cruz Biotechnology	Cat# sc-2314
Rabbit IgG - HRP conjugated	R&D Systems	Cat# HAF008
Goat IgG, conjugated with Alexa Fluor 488	ThermoFisher Scientific	Cat# A-11055
Chemicals, Peptides, and Recombinant Proteins		
0.25% Trypsin	ThermoFisher Scientific	Cat# 25200056
2-deoxy glucose	Sigma	Cat# D6134
Acetone	Sigma	Cat# 24201
Adam-MC cell viability stain	NanoEnTek	Cat# ADR-1000
Amersham ECL Western Blotting Detection Reagent	Sigma	Cat# GERPN2209
Antimycin A	Sigma	Cat# A8674
BSA	Sigma	Cat# A7906
Collagen I	Sigma	Cat# C4243
Collagenase A	Roche	Cat# 11088793001
Collagenase/Dispase	Roche	Cat# 10269638001
cOmplete™ Protease Inhibitor Cocktail	Roche	Cat# 11697498001
Dnase I	Roche	Cat# 11284932001
Donkey Serum	Sigma	Cat# 566460
Dynabeads coated with sheep anti-rat IgG	ThermoFisher Scientific	Cat# 11035
Endothelial cell growth supplement	Sigma	Cat# E2759
F12 HAM nutrient mixture	Sigma	Cat# 51651C
FBS	Gibco	Cat# 10270106
FCCP	Sigma	Cat# C2920
Glucose	Sigma	Cat# G8769
HBSS	ThermoFisher Scientific	Cat# 14175129
Heparin	Sigma	Cat# H3149
HEPES	Sigma	Cat# 15630-056
Low-glucose DMEM	Sigma	Cat# D6046
Mitomycin C	Sigma	Cat# D6046
MitoTracker Red CMXRos	ThermoFisher Scientific	Cat# M7512
NE-PER™ Nuclear and Cytoplasmic Extraction Reagents	ThermoFisher Scientific	Cat# 78833
Non-essential amino acids	Sigma	Cat# M7145
NuPAGE™ 3-8% Tris-Acetate Protein Gels	ThermoFisher Scientific	Cat# EA0375BOX
NuPAGE™ 4-12% Bis-Tris Protein Gels	ThermoFisher Scientific	Cat# NP0321BOX
NuPAGE™ LDS Sample Buffer (4X)	ThermoFisher Scientific	Cat# NP0007
NuPAGE™ Sample Reducing Agent (10X)	ThermoFisher Scientific	Cat# NP0004
Oligomycin	Sigma	Cat# O4876

REAGENT/RESOURCE	REFERENCE/SOURCE	IDENTIFIER or CATAOGUE NUMBER
Pierce™ ECL Western Blotting Substrate	ThermoFisher Scientific	Cat# 32209
Power Blotter Select Transfer Stacks, PVDF	ThermoFisher Scientific	Cat# PB5310
ProLong Diamond Antifade with DAPI	ThermoFisher Scientific	Cat# P36962
Puromycin Dihydrochloride	Sigma	Cat# P8833
Rotenone	Sigma	Cat# R8875
Seahorse XF base medium	Agilent	Cat# 103334-100
Sodium pyruvate	ThermoFisher Scientific	Cat# 11360039
Trans-Blot® Turbo™ Mini PVDF Transfer Packs	Bio-Rad	Cat# 1704156
Triton-X100	Sigma	Cat# T8787
Tween	VWR Chemicals	Cat# 9005-64-5
**Critical Commercial Assays**		
RealTime-Glo™ MT Cell Viability Assay	Promega	Cat# G9711
RNeasy isolation kit	Qiagen	Cat# 74106
SuperScript III reverse transcriptase kit	ThermoFisher Scientific	Cat# 18080093
Pierce™ BCA Protein Assay Kit	ThermoFisher Scientific	Cat# 23227
Glucose Uptake-Glo™ Assay	Promega	Cat# J1341
Succinate Dehydrogenase Activity Assay Kit	Abcam	Cat# ab228560
Succinate Assay Kit	Abcam	Cat# ab204718
**Experimental Models: Cell Lines**		
Primary Microvascular murine EC	This paper	
**Experimental Models: Organisms/Strains**		
C57/BL6 WT mouse	In house breeding	
**Oligonucleotides**		
Arginase II primer fwd ACCAGGAACTGGCTGAAGTG	[100]	
Arginase II primer rev TGAGCATCAACCCAGATGAC		
β-actin primer fwd AGAGGGAAATCGTGCGTGAC	[101]	
β-actin primer rev CAATAGTGATGACCTGGCCGT		
β-actin probe /FAM/CACTGCCGCATCCTCTTCCTCCC/BHQa-Q/		
BNIP3 primer fwd GACGAAGTAGCTCCAAGAGTTCTCA	[47]	
BNIP3 primer rev CTATTTCAGCTCTGTTGGTATCTTGTG		
Epas1 primer fwd GTCCGAAGGAAGCTGATGG	[102]	
Epas1 primer rev TCTATGAGTTGGCTCATGAGTTG		
Epas1 probe /FAM/CCACCTGGACAAAGCCTCCATCAT/36-TAMSp/		
GLUT-1 primer fwd GGGCATGTGCTTCCAGTATGT	[41]	
GLUT-1 primer rev ACGAGGAGCACCGTGAAGAT		
GLUT-1 probe /FAM/CAACTGTGCGGCCCCTACGTCTTC/BHQ/		
HIF-1α primer fwd GGTGCTGGTGTCCAAAATGTAG	[103]	
HIF-1α primer rev ATGGGTCTAGAGAGATAGCTCCACA		
HIF-1α probe /FAM/CCTGTTGGTTGCGCAGCAAGCATT/36-TAMSp/		
iNOS primer fwd ACCCTAAGAGTCACCAAAATGGC	[41]	
iNOS primer rev TTGATCCTCACATACTGTGGACG		
LDH-A primer fwd TGTCTCCAGCAAAGACTACTGT	[41]	
LDH-A primer rev GACTGTACTTGACAATGTTGGGA		
PGK primer fwd CAAATTTGATGAGAATGCCAAGACT	[41]	
PGK primer rev TTCTTGCTGCTCTCAGTACCACA		
PGK probe /FAM/TATACCTGCTGGCTGGATGGGCTTGGACT/BHQa-Q/		
VEGF primer fwd TGAAGCCCTGGAGTGCGT	[41]	
VEGF primer rev AGGTTTGATCCGCATGATCTG		
VEGF probe /FAM/CCCACGTCAGAGAGCAACATCACCA/BHQa-Q/		
**Software and Algorithms**		
ImageJ	[104]	https://imagej.nih.gov/ij/
Mars Version NO. 3.32R	BMG Labtech	https://www.bmglabtech.com/mars-data-analysis-software/

REAGENT/RESOURCE	REFERENCE/SOURCE	IDENTIFIER or CATAOGUE NUMBER
Wave 2.6	Agilent Technologies, Inc.	https://www.agilent.com/en/products/cell-analysis/cell-analysis-software/data-analysis/wave-desktop-2-6
Prism 9	GraphPad Software, Inc.	https://www.graphpad.com/scientific-software/prism/
Other		
FLUOstar Omega microplate reader	BMG Labtech	https://www.bmglabtech.com/fluostar-omega/
Whitley H35 Hypoxystation	Don Whitley Scientific	https://www.dwscientific.com/whitley-hypoxic-workstations/h35-hypoxystation
Seahorse XFe96 Analyzer	Agilent Technologies, Inc.	https://www.agilent.com/en/products/cell-analysis/seahorse-analyzers/seahorse-xfe96-analyzer
Seahorse XF24-3 Analyzer	Agilent Technologies, Inc.	Discontinued
Trans-Blot Turbo Transfer System	Bio-Rad	Cat# 1704150
Power Blotter XL System	ThermoFisher Scientific	Cat# PB0013
